# Machine learning models reveal microbial signatures in healthy human tissues, challenging the sterility of human organs

**DOI:** 10.3389/fmicb.2024.1512304

**Published:** 2025-01-27

**Authors:** Anargyros Skoulakis, Giorgos Skoufos, Armen Ovsepian, Artemis G. Hatzigeorgiou

**Affiliations:** ^1^DIANA-Lab, Department of Computer Science and Biomedical Informatics, University of Thessaly, Lamia, Greece; ^2^Hellenic Pasteur Institute, Athens, Greece

**Keywords:** human tissues, human organs, tissues, microbiome, microbial communities, microorganisms, microbial signatures, machine learning

## Abstract

**Background:**

The presence of microbes within healthy human internal organs still remains under question. Our study endeavors to discern microbial signatures within normal human internal tissues using data from the Genotype-Tissue Expression (GTEx) consortium. Machine learning (ML) models were developed to classify each tissue type based solely on microbial profiles, with the identification of tissue-specific microbial signatures suggesting the presence of distinct microbial communities inside tissues.

**Methods:**

We analyzed 13,871 normal RNA-seq samples from 28 tissues obtained from the GTEx consortium. Unaligned sequencing reads with the human genome were processed using AGAMEMNON, an algorithm for metagenomic microbial quantification, with a reference database comprising bacterial, archaeal, and viral genomes, alongside fungal transcriptomes. Gradient-boosting ML models were trained to classify each tissue against all others based on its microbial profile. To validate the findings, we analyzed 38 healthy living tissue samples (samples from healthy tissues obtained from living individuals, not deceased) from an independent study, as the GTEx samples were derived from post-mortem biopsies.

**Results:**

Tissue-specific microbial signatures were identified in 11 out of the 28 tissues while the signatures for 8 tissues (Muscle, Heart, Stomach, Colon tissue, Testis, Blood, Liver, and Bladder tissue) demonstrated resilience to *in silico* contamination. The models for Heart, Colon tissue, and Liver displayed high discriminatory performance also in the living dataset, suggesting the presence of a tissue-specific microbiome for these tissues even in a living state. Notably, the most crucial features were the fungus *Sporisorium graminicola* for the heart, the gram-positive bacterium *Flavonifractor plautii* for the colon tissue, and the gram-negative bacterium *Bartonella machadoae* for the liver.

**Conclusion:**

The presence of tissue-specific microbial signatures in certain tissues suggests that these organs are not devoid of microorganisms even in healthy conditions and probably they harbor low-biomass microbial communities unique to each tissue. The discoveries presented here confront the enduring dogma positing the sterility of internal tissues, yet further validation through controlled laboratory experiments is imperative to substantiate this hypothesis. Exploring the microbiome of internal tissues holds promise for elucidating the pathophysiology underlying both health and a spectrum of diseases, including sepsis, inflammation, and cancer.

## 1 Introduction

Microbes exhibit a remarkable spectrum of functions and capabilities that enable them to colonize diverse and extreme habitats (Merino et al., 2019). The human body serves as a host for a vast array of microbes with varied functionalities (Ursell et al., 2012). Our understanding regarding interactions between humans and microbes has evolved through the progression of sequencing technologies. Pioneering this transformative trajectory was the NIH Human Microbiome Project (HMP), which revealed the complex interplay between human and microbial cells (The Human Microbiome Project Consortium, 2012; The Human Microbiome Project Consortium, 2012; The Integrative HMP (iHMP) Research Network Consortium, 2019). Recent advancements have prompted a reevaluation of long-standing beliefs, showcased by the dismissal of the traditional notion that healthy human lung and vagina are sterile environments (Natalini et al., 2023; Chen et al., 2021).

The predominant focus of microbiome studies has been on characterizing microbial communities in easily accessible sampling sites, including the human intestinal tract, the oral cavity, and the skin. Internal tissues, less accessible by conventional sampling methods, have historically been presumed to be devoid of microorganisms due to protective layers of epithelial and endothelial tissues. However, this assumption has been challenged, particularly in settings such as cancer, where various studies have revealed the presence of tumor-specific microbial sequences within human tumors, offering a potential new avenue for cancer diagnosis (Poore et al., 2020; Dohlman et al., 2021; Narunsky-Haziza et al., 2022; Nejman et al., 2020; Riquelme et al., 2019; Ghaddar et al., 2022; Aykut et al., 2019). As the exploration of microbes inside internal tissues has primarily been conducted in the context of pathological conditions like cancer, fundamental questions regarding healthy status persist. Are there microbial residents within human tissues engaged in a commensal relationship with human cells? What is the role of their presence? Can these microbial signatures trigger an immunological response or serve as diagnostic markers for organ health?

Mahmoudabadi et al. observed a notable presence of bacterial genera in tumors that is also detectable and similar in adjacent tumor-free tissues (Mahmoudabadi et al., 2022). This suggests that tumor microbiomes may partially originate from neighboring normal tissues, indicating that, under healthy conditions, internal tissues host a microbiome. Hieken et al. also detected microbial communities in aseptically collected human breast tissues in benign and malignant conditions (Hieken et al., 2016). Moreover, investigations in germ-free mice have unveiled microbiomes within various organs, including the brain, muscle, adipose tissue, liver, and heart. This challenges the conventional notion that the internal organs of mammals are devoid of microbial presence (Lluch et al., 2015).

**Figure 1:**
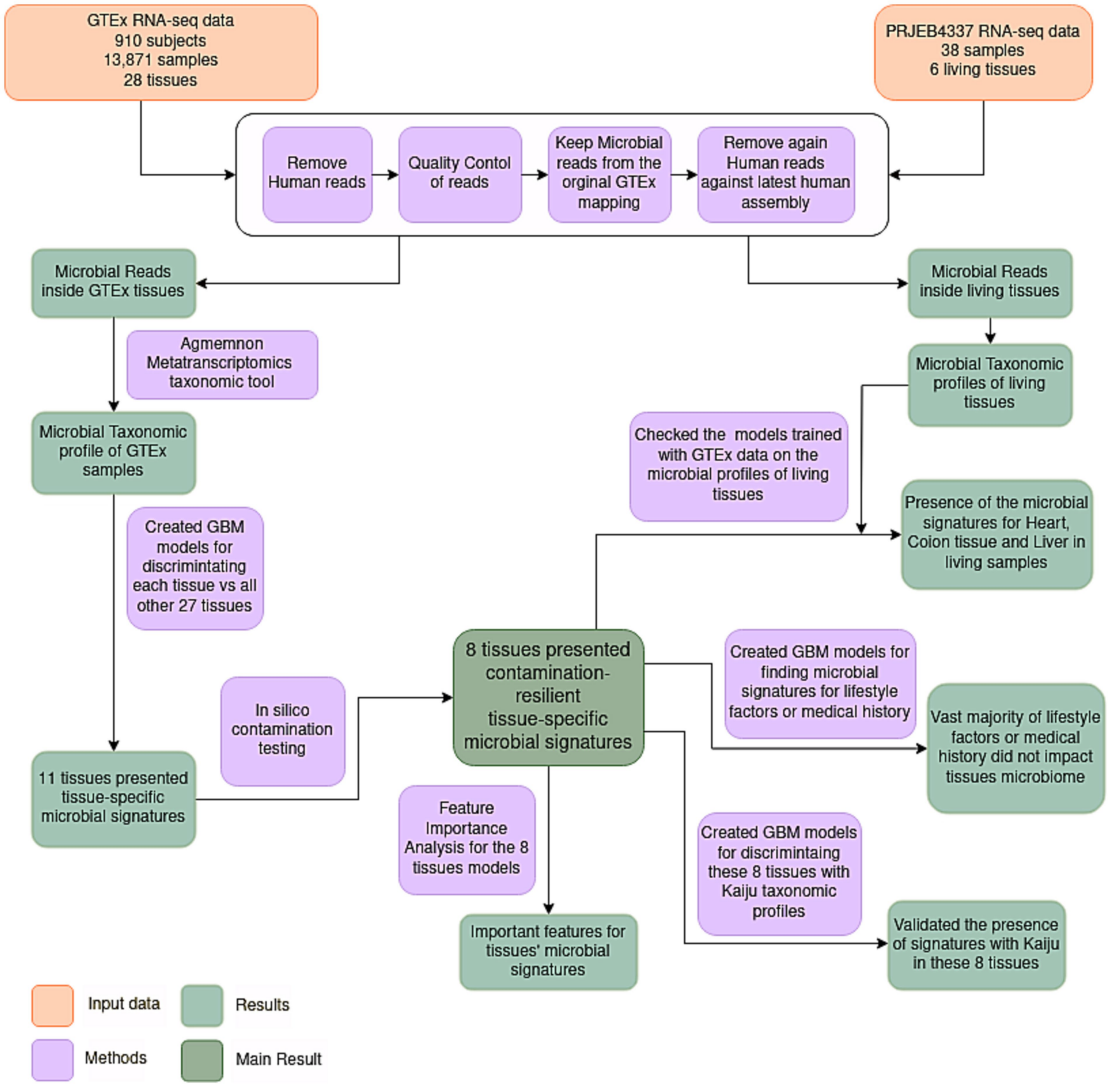
Flowchart illustrating the employed methodology. RNA-seq samples were quality controlled and pre-processed. Subsequently, microbial reads were assigned to specific microbial species utilizing AGAMEMNON, a very accurate metagenomics and metatranscriptomics quantification analysis suite. The microbial taxonomic profiles of samples originating from diverse tissues were then utilized to discriminate the different tissues using Gradient Boosting ML models. Eleven tissues presented tissue-specific microbial signatures, capable of discriminating these tissues against all the others. Following an *in-silico* contamination analysis, eight tissues presented contamination-resilient microbial signatures, underscoring the potential existence of tissue-specific microbiomes in these tissues. Additionally, the models of these 8 tissues, trained in GTEx data, were further tested on living samples (NCBI project PRJEB4337), and 3 living tissues (heart, colon and liver) were discriminated based on their microbial signatures. Furthermore, these 8 tissues were further analyzed to find the most important features of these signatures. An evaluation of the potential influence of various lifestyle factors and medical history was conducted on the identified signatures. Lastly, the presence of tissue-specific models was further validated using Kaiju, a different metatranscriptomics taxonomic tool.

To elucidate the microbiome inside healthy human tissues, we conducted a re-analysis of RNA-seq data derived from Genotype-Tissue Expression (GTEx) consortium (Lonsdale et al., 2013). Originally designed to explore variations in gene expression within healthy human tissues, GTEx’s extensive repository contains samples exclusively derived from healthy specimens. Acknowledging certain limitations within the GTEx analysis pipeline, such as the employment of a poly-A selection protocol and the reliance on post-mortem biopsy samples, it remains noteworthy that the GTEx consortium provides the most extensive and analytically robust dataset about RNA expression within healthy human tissues. To date, GTEx sequencing data have not been explored from a microbiome perspective. Here, we present what, to our knowledge, stands as the inaugural and comprehensive healthy human tissue microbiome dataset. Leveraging ML models, we identified microbial signatures capable of discriminating among various tissue types and investigated the potential influence of phenotypic traits (e.g., age, sex, BMI) on these signatures. The presence of tissue-specific microbial signatures in certain tissues would suggest that these organs are not devoid of microorganisms even in healthy conditions, thus challenging the long-standing dogma of the sterility of internal tissues. The workflow of the study is shown in Figure 1.

## 2 Materials and methods

### 2.1 GTEx data accession

The data used for the analyzes described in this manuscript were obtained from dbGaP accession number phs000424.v9.p2 on 04/01/2023. All GTEx RNA-seq data and matched samples protected metadata were accessed via NHGRI Analysis Visualization and Informatics Lab-space (AnVIL).^1^ Details of how these data were downloaded, are comprehensively described in the AnVIL site.^2^ For bulk data acquisition, custom Snakemake files were employed. Due to constraints in storage capacity, an approach was implemented wherein each sample BAM files were downloaded, and only the unmapped reads [as was originally mapped by GTEx pipeline using STAR (version 2.5.3a) (Dobin et al., 2013)] were retained using samtools (version 1.10) (Danecek et al., 2021).

### 2.2 GTEx sample and subject QC

The original GTEx dataset initially comprised 17,350 RNA-seq samples derived from 948 subjects, all of which had successfully met the GTEx original quality control (QC) criteria. Subsequently, 52 samples were excluded from the analysis due to missing data in critical variables, namely, Genotype or Expression Batch ID, Date of genotype or expression batch, and Total Ischemic time. The dataset was further refined to encompass only 14,478 samples originating from tissues preserved in the PAXgene tissue fixative solution, specifically categorized under “PAXgene” and “Whole Blood:PAXgene” in the “current_material_type” variable. Furthermore, an additional refinement process involved excluding 32 samples originating from tissue sites with fewer than 20 samples, specifically samples from the following tissues sites: Kidney Medulla, Fallopian Tube, Cervix Extocervix, and Cervix Endocervix were removed. Additionally, 3 samples possessing an RNA Integrity Number (RIN) less than 5 were excluded, along with 139 samples with Total Ischemic time exceeding 1,440 min (24 h). Following these rigorous sample quality control measures, a total of 14,304 samples from 942 subjects and spanning 28 different tissues remained in the dataset.

Following, quality control of the subjects was performed. Nine subjects were initially excluded due to their ineligibility based on GTEx original criteria. Seven subjects were then removed from the dataset as they had a current cancer diagnosis, and 16 subjects were excluded due to a history of cancer diagnosis within the past 5 years. All the samples from the excluded subjects were removed. Following this comprehensive sample and subject quality control process, the resulting dataset comprised 13,871 RNA-seq samples originating from 28 distinct tissues (refer to Supplementary File S1) and derived from 910 subjects. The quality control procedures were implemented utilizing custom Python scripts, available on the corresponding GitHub repository.

### 2.3 Isolation of unmapped reads and quality control

For each sample, sequencing reads that failed to align with the human reference genome, as indicated by the mapping information in the raw BAM files obtained from GTEx,^3^ were selectively retained. To isolate the unmapped reads where both paired reads were unaligned, and to eliminate reads classified as non-primary alignments, bioinformatic tool Samtools was employed using the arguments “-f 12 -F 256.”

The unmapped reads underwent a comprehensive quality control process, involving the exclusion of reads with a length shorter than 35 nucleotides (nt), given that the GTEx normal sequencing length was 76 nt. Additionally, steps were taken to remove adapters and perform quality trimming with a threshold at Phred quality score of 15. These quality control procedures were executed using Atropos (version 1.1.31) (Didion et al., 2017). To streamline and automate this sequence of steps, custom Snakemake scripts were implemented, available on the corresponding GitHub repository.

### 2.4 Taxonomic assignment using AGAMEMNON

To construct the reference database utilized for the taxonomic algorithm AGAMEMNON (Skoufos et al., 2022) (version 0.1.0), a custom Bash script was employed on 02/14/2023 to download microbial genomes from RefSeq. The dataset comprised all bacterial representative or reference genomes with complete genome assembly level (4,034 bacterial genomes), all archaeal genomes with complete genome assembly level (489 archaea genomes), and all viral genomes with complete genome assembly level (11,259 viral genomes). Furthermore, fungal transcriptomes from representative or reference genomes with complete genome or chromosome assembly levels (81 fungal transcriptomes) were also included in the reference database. In the case of fungi, the transcriptome data was specifically employed to account for the intricacies of the splicing process. This approach ensures a more comprehensive representation of fungal genomic information, taking into consideration the variations introduced during the splicing of transcripts.

The quality-controlled non-human reads of each sample underwent mapping against the custom reference database described above, using the Puffaligner algorithm (Almodaresi et al., 2021). For Puffaligner, the flag “--noOrphans” was used in order to discard the orphans reads. The term “orphan” refers to one end of paired-end read that is confidently aligned to some genomic position, but for which the other read end is not jointly aligned nearby (and paired). Puffaligner aligns the reads to the compiled microbial genomes, enabling the identification of reads of microbial origin within the samples. Puffaligner is a fast, sensitive and accurate aligner based on a compacted sequence graph and is meticulously crafted to embody a dual emphasis on high sensitivity in alignment tasks and efficient computational performance. Its design capitalizes on the utilization of a colored compacted de Bruijn graph to efficiently identify and factor out recurring sub-sequences within the reference.

As highlighted by Gihawi et al. (2023), in order to be sure that no human read had succeeded to infiltrate in our analysis, we remapped the reads that were classified by Puffaligner as reads of microbial origin to the most recent human genome assembly (T2T-CHM13v2.0 from T2T Consortium) using bowtie2 (version 2.2.3) (Langmead et al., 2019; Langmead and Salzberg, 2012) with the “preset” parameters of “--very-sensitive” and kept all the reads that were not mapped concordantly. Custom snakemake scrips were used to automate the process. Then by using only the remaining reads, we analyzed them with AGAMEMNON (a changed version, deposited in the github repo of the present study) to find the microbial profile of each sample. AGAMEMNON represents a metagenomics and metatranscriptomics algorithm, notable for its integration of a time and space-efficient indexing scheme. This feature facilitates rapid pattern matching, allowing for the efficient indexing and analysis of extensive datasets using commonly available computational resources. In the abundance estimation step, the primary approach relies on the expectation maximization (EM) algorithm. The goal is to maximize the likelihood of observed reads by iteratively adjusting the abundance values linked to various taxa. At last, after running AGAMEMNON, the taxonomic profile of each sample was generated.

### 2.5 Diversity metrics and core microbiome per tissue

The taxonomic profiles generated by AGAMEMNON at the species level were employed to calculate the microbiome richness of each sample. Microbiome richness was defined as the count of species with non-zero abundance in each sample. To compute the Shannon diversity index, the “diversity” function from the R package vegan (version 2.6.4) was utilized.

The core microbiome for each tissue was defined by considering all species present in at least 10% of the respective tissue’s samples. The 10% threshold was chosen to exclude species identified in a limited number of samples, that may be indicative of opportunistic infections, and to retain only species that are consistently present in tissue samples. Given the inherent diversity and dynamic nature of the microbiome, a relatively low threshold (10%) was applied to avoid excluding too many species. For comparisons of the core microbiome across tissues, UpSet plots were generated using the R library UpSet (version 1.4.0).

### 2.6 Normalization of taxonomic profiles

To address biases, particularly due to differences in sequencing depth among samples, we employed Cumulative Sum Scaling (CSS) normalization in the taxonomic profiles (Paulson et al., 2013). CSS, functioning as a median-like quantile normalization method, corrects for variations in sampling depth or library size. Unlike standard relative abundance normalization, which rescales all samples to a uniform total sum (e.g., 100%), CSS retains variability in total counts across samples. This normalization method adjusts samples based on a subset (quartile) of lower-abundance taxa that remain relatively constant and independent, mitigating the impact of high-abundance taxa that may dominate a study. For CSS normalization, the metagenomeSeq library (version 1.40.0) in R was employed. This normalization approach was also applied to normalize taxonomic profiles at the genus level and functional profiles generated by HUMAnN 3 (Beghini et al., 2021).

### 2.7 ML models 1vsAllOther27tissues

To discern potential biological significance within the taxonomic profiles of samples, we endeavored to construct ML models for each tissue to discriminate it from all the other 27 tissues. We trained stochastic gradient boosting machine (GBM) learning models, known for their efficacy in classification tasks and resilience to imbalanced datasets (Friedman, 2001). The models were implemented and fine-tuned using the R libraries GBM (version 2.1.8.1), Caret (version 6.0.94), and PRROC (version 1.3.1) (for calculating AUROC and AUPR values). The training and testing phases occurred on distinct, randomly selected, stratified sampling splits of 70 and 30% of the data, respectively, with a fixed random seed for reproducibility. CSS normalization was performed separately in each split. After normalization of each split, only the total core species of the 28 tissues were retained for training the model as we were interested in the signatures present in the core microbiome within tissues.

Two-fold cross-validation and grid search optimization were applied to tune GBM parameters, including interaction depth (1–3) and the number of trees (50–150), while keeping the learning rate at 0.1 and minimum observations per node at 3. Up-sampling of the minority class was used during training to address class imbalance. Final model performance metrics (AUROC and AUPR) were calculated on the unseen test set, with 100 iterations performed per tissue model to compute mean values and 95% confidence intervals. Relative AUPR, defined as the ratio of AUPR (model) to AUPR (random), was used as a normalized performance metric. For computational efficiency, ML scripts utilized 10 cores with the R libraries parallel (version 4.2.2) and “doMC” (version 1.3.8). Pearman’s rank correlation was used to assess the relationship between mean AUROC/relative mean AUPR and sample size using the R library stats (version 4.2.2). The same approach was applied at the genera level, using 738 core microbial genera across 28 tissues as features.

### 2.8 *In silico* contamination

**Figure 2:**
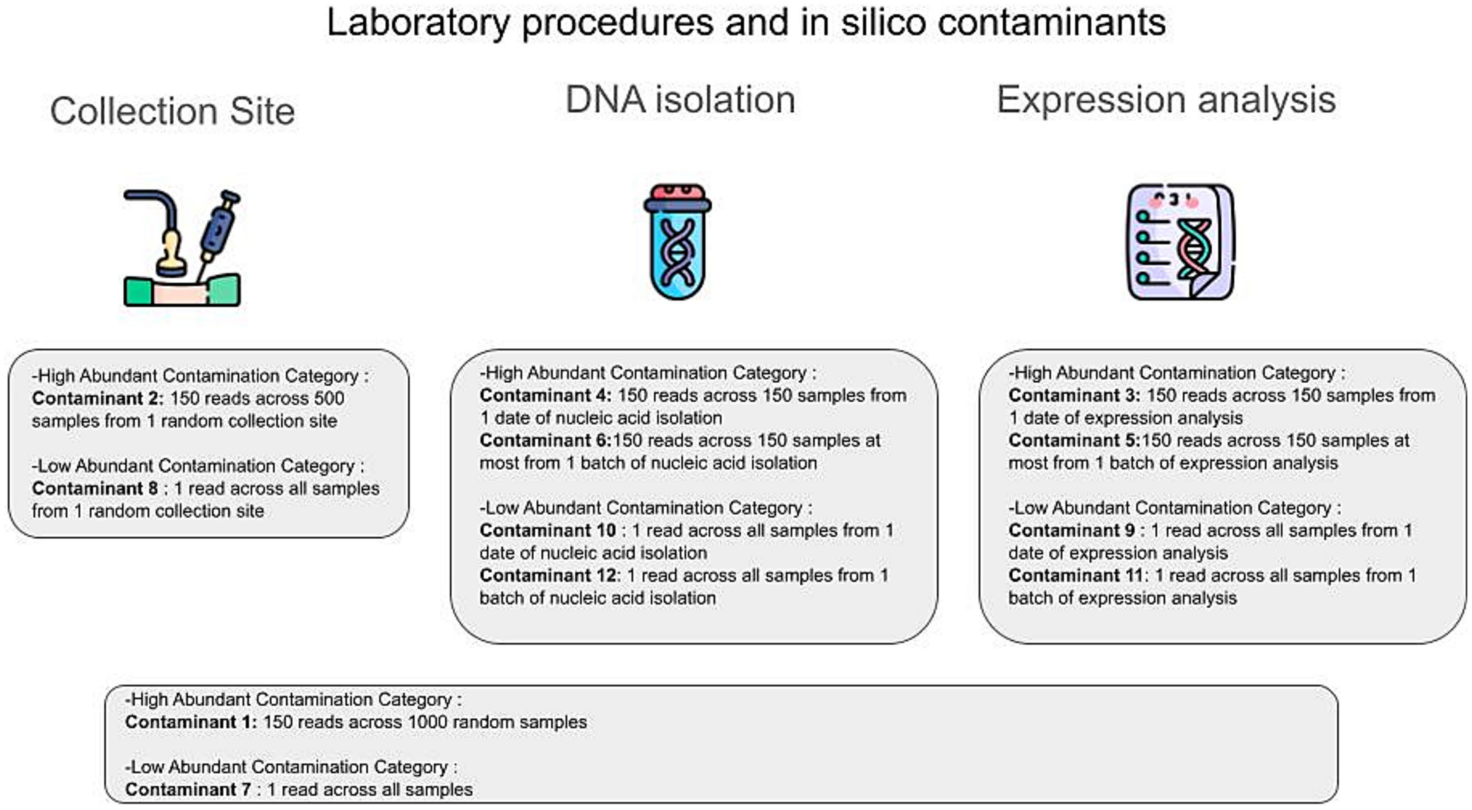
The different types of *in silico* contaminants. *In silico* contaminants can be classified into two main categories: high abundance contamination and low abundance contamination. The primary routes of contamination in the GTEx original analysis protocol include the collection site, the DNA isolation step (DNA/RNA isolation procedure), and the expression analysis step (RNA sequencing procedure). At each step, we introduced different types of *in silico* contaminants to mitigate possible contamination scenarios.

For the *in silico* contamination approach, we introduced 12 distinct contaminants into the dataset, simulating two categories: high-volume contaminants, characterized by their high volume (high number of reads attributed to them) on a small subset of samples, positing that their detection would be possible if they affected a larger sample pool; and low-volume contaminants, which, although affecting a greater number of samples, did so at lower volumes (small number of reads attributed to them), thereby evading their detection. This included six contaminants of each category, as illustrated in Figure 2. Contaminants were added post-CSS normalization, and 100 iterations were performed, randomly selecting contaminated samples in each iteration using a custom Python script. Gradient Boosting Machine (GBM) models were constructed for the 11 tissues with tissue-specific microbiomes, using the core microbiome (1,612 microbial species plus 12 contaminants) to distinguish each tissue from the other 10. Additionally, 100 uncontaminated GBM models were created for comparison. Feature importance was assessed using GBM and Caret, with contributions calculated as the percentage of the total feature importance score (by dividing its importance score by the sum of all features’ importance scores for the given model). Wilcoxon test was applied to compare AUROC and AUPR values between contaminated and uncontaminated models across the 11 tissues using the R library stats.

### 2.9 ML models 1vs7 tissues

To focus on the signatures identified within the 8 contamination-resilient tissues (Blood, Testis, Colon, Stomach, Muscle, Bladder, Liver, and Heart), we reconstructed GBM models using samples exclusively from these tissues. These models utilized the concatenated core microbiome of the 8 tissues, consisting of 1,597 species for species-level models. The configurations mirrored those described in the section ML models 1vsAllOther27tissues. Each model was executed 100 times to compute the mean AUROC, AUPR, and margins of error. Feature importance scores were estimated using the first iteration of the models, highlighting differences in microbial compositions across these tissues. The same methodology was applied at the genus level, using 695 genera as features from the combined core microbiome of the 8 tissues.

### 2.10 ML models with Kaiju

To validate the robustness of findings for the 8 tissue-specific signatures, we utilized an alternative taxonomic tool, Kaiju (Menzel et al., 2016) (version 1.9.2), to identify the taxonomic profiles of samples from the 8 contamination-resilient tissues. Using QCed reads (post-Atropos processing), Kaiju classified sequencing reads by comparing their translated amino acid sequences against the microbial subset of the NCBI BLAST non-redundant protein database (nr), including fungi and microbial eukaryotes. The database was downloaded on 03/30/2023 from https://bioinformatics-centre.github.io/kaiju/downloads.html. The kaiju2table tool converted the outputs into summary tables at the species level.

Based on these taxonomic profiles, GBM models were reconstructed for each tissue to discriminate it from the other 7 tissues, following the methodology described earlier. The features for these models included the concatenated core microbiome of the 8 tissues, as identified by Kaiju, comprising 1,864 microbial species. Each model was executed 100 times to compute mean AUROC, AUPR, and margins of error. Feature importance scores were estimated using a single iteration, as previously described.

### 2.11 Functional assignment using HUMAnN 3

To characterize the functional profile of samples from the GTEx consortium, reads that were identified as microbial by AGAMEMNON were analyzed using HUMAnN 3 (version 3.6.1) for profiling the abundance of microbial genes. HUMAnN 3 is a method designed to efficiently and accurately conduct functional profiling in metagenomic or metatranscriptomic sequencing data. Due to constraints in computational resources and time, HUMAnN 3 was executed with the option “--bypass-nucleotide-search” to skip all alignment steps before the translated search. For this translated search, the full UniRef90 database (version 201901b) served as the reference.

Subsequently, the functional profiles of all samples were concatenated and normalized using CSS normalization. GBM models were then created following the approach described previously, utilizing the 1-vs-7 tissues strategy and incorporating only the core gene repertoire. This repertoire consisted of genes present in at least 10% of each tissue’s samples, amounting to 1,831 different microbial genes across the 8 tissues.

For models integrating both functional and taxonomic profiles, the core gene repertoire (1,831 genes) and the core species microbiome (1,597 species) were utilized in the creation of GBM models. Each component was separately normalized using CSS normalization, and then the normalized genes’ and species’ taxonomy profiles were merged for the training and testing datasets. The GBM models were constructed using the same parameters as described earlier. To calculate the mean AUROC and AUPR, along with their margins of error, 100 iterations of each model were generated.

### 2.12 Factors associated with tissue microbiome

To investigate the influence of various traits [age, Body Mass Index (BMI), smoking status, drinking status, ancestry], as well as disease history (hypertension history, ischemic heart history, diabetes II history, diabetes I history, arthritis history, seizures history, schizophrenia history, rheumatoid arthritis history, liver disease history, dialysis treatment, depression history, COPD or CLRD history, cerebrovascular disease history, asthma history, Alzheimer or dementia history), on the core microbiome of the 8 tissues with contamination-resilient tissue-specific microbiomes, GBM models were created for each trait and tissue combination with aim to discriminate in each tissue the samples with the specific trait from the samples without this trait. Tissues with fewer than 20 samples within each different group of specific trait/disease were excluded due to insufficient data for model creation.

For continuous traits such as age and BMI, GBM models were constructed using 5-fold cross-validation and Root Mean Squared Error (RMSE) was used to select the optimal model with the smallest RMSE value. Mean Absolute Error (MAE) and R-squared were calculated using the “postResample” function from the Caret package. The mean RMSE, MAE, and R-squared were computed across 100 iterations of each model.

For categorical traits (smoking, drinking, sex, and ancestry), as well as models for disease history, GBM models were developed similarly to tissue models but with 4-fold cross-validation to mitigate overfitting. Mean AUROC and mean AUPR were computed across 100 iterations of each model. For the ancestry trait, samples with ancestries other than “White” or “Black or African American” were removed due to limited representation.

### 2.13 Validating significant ML models with living samples

To validate the previously developed 1-vs-7 tissue models in living tissues, we analyzed RNA-seq data from the 8 contamination-resilient tissues using samples from NCBI Bioproject PRJEB4337, which contains living tissue samples from the Swedish Biobank. Only samples corresponding to these tissues were retained, although this project lacked data for muscle tissue and blood. The RNA-seq data were generated using a poly-A selection protocol and processed consistently with GTEx samples.

First, the fastq files were aligned to the human genome using STAR (version 2.7.10b), and unmapped reads were isolated and quality-controlled using Atropos with the same settings. These QCed reads were mapped to the microbial database using Puffaligner, remapped to the latest human genome with Bowtie2, and finally analyzed taxonomically using Agamemnon, ensuring identical processing pipelines for GTEx and PRJEB4337 samples.

Next, the original 1-vs-7 tissue models (100 iterations per tissue) developed using GTEx data were tested on the 38 PRJEB4337 living samples. Performance metrics, including AUROC and AUPR, were calculated using the PRROC library. To confirm that the high performance observed was due to tissue-specific microbial signatures, 100 iterations of random models were created by shuffling tissue labels in the GTEx dataset. These random models were then tested on the living dataset, and their performance was compared with the original models using a Wilcoxon test. This comparison ensured that observed performance was driven by tissue-specific microbial signatures rather than random chance. Additionally, samples from PRJEB4337 were analyzed using the Kaiju tool to generate microbial profiles, following the same pipeline used for GTEx data. The GBM models based on Kaiju profiles of GTEx data were tested on the PRJEB4337 samples, with 100 iterations executed for performance evaluation.

## 3 Results

### 3.1 Low-biomass microbial RNA is detected in GTEx samples

A total of 13,871 RNA-seq samples originating from 28 distinct tissues (e.g., Lung, Liver, Pituitary, Blood Vessel, Thyroid, Skin, Salivary Gland, Esophagus, Heart, Muscle, Pancreas, Adipose Tissue, Vagina, Blood, Ovary, Spleen, Prostate, Adrenal Gland, Nerve, Stomach tissue, Colon tissue, Testis, Brain, Breast, Uterus, Small Intestine tissue, Kidney, Bladder tissue) within the GTEx consortium have been utilized. All samples were designated as non-diseased and free of pathology, as the specimens were reviewed by a panel of 2–3 pathologists and any specimen found with an incidental finding had been systematically excluded.

**Figure 3:**
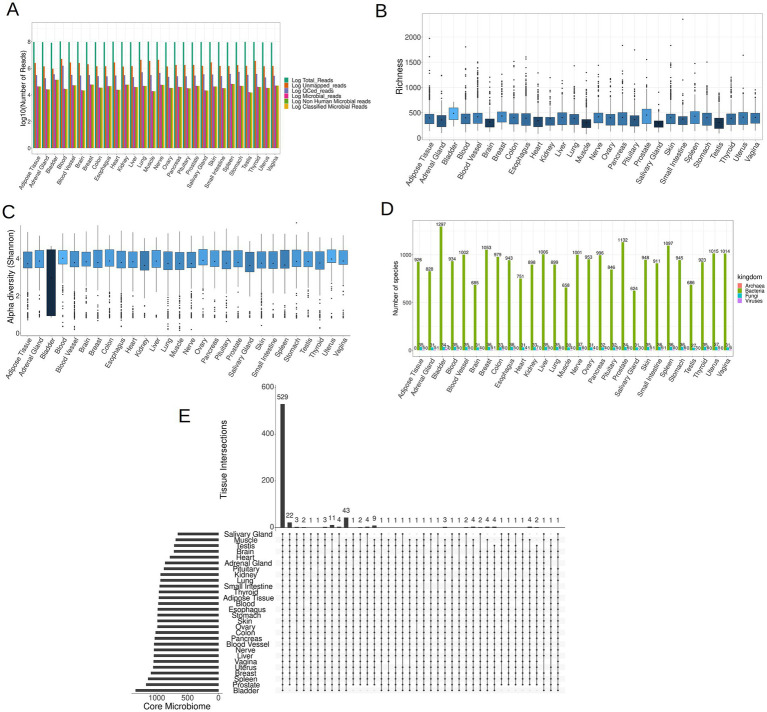
**(A)** The mean of reads (log10 space) at each step of the analysis, including raw reads, unmapped to human genome reads, QCed reads, microbial reads, non-human microbial reads and lastly classified microbial reads. **(B)** Boxplots illustrating the microbial richness of the samples across 28 tissues (the darker shades of blue correspond to lower mean values, while brighter shades indicate higher mean values). **(C)** Boxplots depicting the microbial Shannon index of samples in each tissue. **(D)** The distribution of the core microbiome per tissue in the four kingdoms of life. **(E)** An UpSet plot illustrating the shared species present in the core microbiome across all tissues, comprising a total of 1,708 species.

Following the removal of reads of human origin, the remaining reads were aligned against an extensive database comprising 4,034 bacterial, 489 archaeal, 11,259 viral, and 81 fungal entities. The microbial composition of each sample was determined utilizing AGAMEMNON. AGAMEMNON provides precise genus, species, and strain abundances through an efficient indexing scheme for rapid pattern matching, facilitating analysis of extensive datasets using common computational resources. The abundance estimation employs expectation maximization algorithm and targets maximizing the likelihood of the observed reads by gradually altering the abundance value associated to different taxa. On average, out of ~93 × 10^6^ raw reads per sample, ~2 × 10^6^ reads (2.35% of the raw reads) did not align to the human genome. After quality control (QC) and filtering of the non-human reads, ~3.5 × 10^5^ reads (0.38% of the raw reads) were preserved. Employing Puffaligner, ~4.3 × 10^4^ (0.046% of the raw reads) were attributed to bacteria, archaea, fungi, or viruses. To eliminate any residual human-associated sequences, a realignment of microbial reads to the most recent human genome assembly (T2T-CHM13v2.0) was executed, resulting in ~4.2 × 10^4^ reads (0.045% of raw reads) remaining. Finally, using AGAMEMNON, all the remaining reads (4.2 × 10^4^ reads, 0.045% of raw reads) were successfully classified into microbial species (Figure 3A). The reported read counts are the mean values across all samples at each step of the analysis.

The bladder exhibited the most pronounced abundance of microbial reads, whereas the adrenal gland, brain, heart, muscle, salivary gland, and testis demonstrated the lowest microbial read counts (Supplementary File S2). In each of the 28 tissues examined, a discernible presence of low-biomass microbial RNA was identified. The microbial communities detected within these tissues may either genuinely inhabit the respective tissues or be a result of potential contamination. As in the case of microbial reads, the bladder exhibited the highest microbial species richness in its microbial community, whereas the brain, heart, muscle, salivary gland, and testis displayed the lowest richness, as depicted in Figure 3B. Notably, the Shannon index of the bladder was markedly lower compared to the rest of the samples (Figure 3C), which arises from the fact that bladder samples demonstrated elevated counts for a few selected species, and comparatively lower counts for the remaining species.

To identify species that are consistently present in each tissue and are not sporadic opportunistic pathogens, we retained species present in at least 10% of samples from each tissue. The retained species are hereafter referred to as the core microbiome of the tissue. Species meeting this criterion for at least one tissue were included in subsequent analyzes, resulting in a total of 1,708 different species across all four kingdoms. As expected, the tissue with the smallest core microbiome was the brain, consisting of 720 species, while the bladder exhibited the most diverse core microbiome, comprising 1,344 species (Figure 3D; Supplementary File S3 for details). Additionally we noticed that across all tissues, bacteria constituted the predominant component, making up over 95% of the core microbiome for each tissue. The core microbiome of each tissue for all four kingdoms, namely bacteria, fungi, viruses and archaea, is documented in Supplementary Files S4–S7, respectively.

Notably, a total of 529 species, accounting for ~31% of the overall core microbiome, were shared across all tissues, showing that tissues share in some extent a common microbiome (Figure 3E). Among fungi, the majority (24 out of 37 species) were present in all tissues, as indicated in Supplementary Figure S1 and Supplementary File S8. However, for bacteria, only 504 out of 1,297 species (38.86%) and for viruses, only Geobacillus virus E2 out of 13 viruses were present in all tissues, as illustrated in Supplementary Figures S2, S3 and Supplementary Files S9, S10, respectively. Archaea were exclusively identified in specific tissues, including the bladder and kidney (species *Methanocaldococcus jannaschii*), the colon and small intestine tissues (species Methanosarcina sp. WH1), and the spleen (species *Methanocaldococcus jannaschii*) (Supplementary File S7).

### 3.2 Eight tissues harbor specific microbial signatures at species level

**Figure 4:**
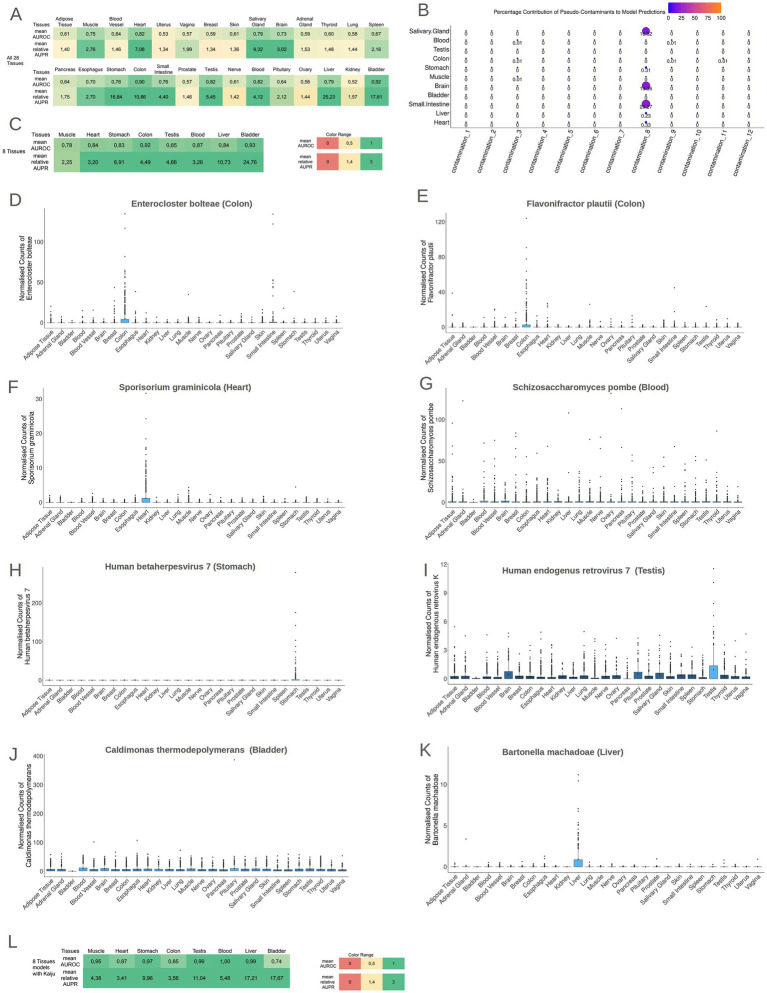
**(A)** The performance of the models (AUROC and relative AUPR) for all tissues in the models 1TissuevsAll27OtherTissues. It is evident that 11 tissues presented high AUROC and AUPR values. **(B)** A bubble plot showing the mean importance score of each contamination type in the 11 tissues models. The contamination 8 plays an important role in small intestine, brain and salivary gland models. **(C)** The performance of the models (AUROC and relative AUPR) for the tissues that presented tissue-specific contamination-resilient microbiome. All eight tissues presented high AUROC and AUPR values in the 1vs7OtherTissues models. **(D–K)** Boxplots of the normalized abundances of the most important features of each model, in the headline of each boxplot is written the species name and in parenthesis the tissue for which this species is the most important feature. **(L)** The performance of the models (AUROC and relative AUPR) for the tissues that presented tissue-specific contamination-resilient microbiome but with the taxonomic profiles produced by Kaiju. All eight tissues presented high AUROC and AUPR values in the 1vs7OtherTissues models even with the employment of a total different taxonomic tool.

While solely the identification of microbiomes within tissues does not warrant the existence of microbial communities within these tissues, the presence of discernible biological footprints within these communities would imply a non-coincidental occurrence of microbes inside these tissues. To explore this, Machine Learning models were employed to detect potential microbial footprints for each tissue. By using *Cum*-Sum Scaling method to normalize microbial profiles across samples, stochastic Gradient Boosting Machine Learning models (GBM models) were trained to distinguish individual tissues from all other tissues. Notably, 11 out of the 28 models (models for Brain, Small Intestine tissue, Liver, Bladder, Muscle, Heart, Salivary Gland, Stomach tissue, Colon tissue, Testis, and Blood) exhibited robust performance (mean AUROC ≥ 0.70 and relative mean AUPR ≥ 1.4) discriminating each specific tissue type from the collective representation of all other tissues, as illustrated in Figure 4A and Supplementary File S11. There was no significant correlation between the sample size and mean AUROC performance (rho = 0.207, *p*-value = 0.28) and the normalized mean AUPR performance (rho = −0.152, *p*-value = 0.43).

The identified microbial signatures based on species abundances within these 11 tissues were further analyzed to investigate if the microbial presence could be attributed to sample contamination. Given that the primary objective of the GTEx initiative was to investigate gene expression, the samples were not processed under sterile conditions. Due to the nature of GTEx experiment protocol, it was inefficient to employ an *in-silico* decontamination approach and to overcome this inefficiency, an alternative strategy was implemented. A randomized *in silico* contamination approach was used to evaluate the potential impact of systematic contamination on the performance of the models. To scrutinize whether possible contamination could drive the performance of ML models exhibiting high performance, 12 pseudo-contaminants were strategically introduced to the data. These pseudo-contaminants aimed to simulate diverse scenarios of contamination throughout the analytical process, encompassing potential contamination scenarios from the initial sampling to the sequencing procedure.

We retrained the ML models of the 11 tissues presenting high discriminatory power (mean AUROC ≥ 0.70 and relative mean AUPR ≥ 1.4) and compared their performances with and without *in silico* contamination (Supplementary File S12). Only the models of Salivary Gland, Brain, and Small Intestine had significantly higher performance (both mean AUROC values and mean AUPR values) in the contaminated iterations, with *in silico* contaminants playing an important role in the models’ performance. On the other hand, for the rest of the models in the contaminated iterations, models did not rely on the inserted pseudo-contaminants (Figure 4B), therefore, the models for the Salivary Gland, Brain, and Small Intestine tissues were deemed unreliable as their high performance could be the result of contamination. The contamination type, that could potentially influence the performance of these 3 models, is a low volume contamination (contamination type 8) which imitates a contaminant that is systematically present in the samples of a collection site center. Nevertheless, in the rest 8 tissues (Blood, Testis, Colon tissue, Stomach tissue, Muscle, Bladder, Liver, and Heart), the tissue-specific microbial signatures exhibited resilience against potential contamination. The ability of GBM models to discriminate these tissues solely based on their microbial profiles independently of any potential contaminations, underscores the presence of distinctive microbial signatures specific to each of these tissues. To investigate microbial differences among these 8 tissues, GBM models were recreated using data exclusively from these contamination-resilient tissues, distinguishing each tissue from the combined representation of all the other 7 tissues. Remarkably, all 8 tissues demonstrated robust performance in terms of AUROC and relative AUPR (mean AUROC ≥ 0.70 and relative mean AUPR ≥ 1.4), as illustrated in Figure 4C and Supplementary File S13.

Collectively, these results suggest that each of these 8 tissues harbors a distinct and unique microbiome. To assess the biological relevance of these microbial signatures, a detailed examination of the most important features from the 8 models was conducted. Boxplots of the abundance of the most important features across the different tissues are depicted in Figures 4D–K. Supplementary File S14 contains the full catalog of features importance scores for each tissue. In the colon tissue model, the 2 most predominant features were the bacterium *Enterocloster bolteae*, formerly known as *Clostridium bolteae*, a recognized constituent of human feces and *Flavonifractor plautii* (formerly *Eubacterium plautii*), which has been isolated from human feces, blood, intra-abdominal pus, and infected soft tissues in humans (Carlier et al., 2010) (Figures 3D,E). In the case of Heart, the most crucial feature was the fungus *Sporisorium graminicola* and for the Blood model, the 2 most important features were *Schizosaccharomyces pombe* and *Sporisorium graminicola* (Figures 4F,G, respectively). These two fungal species are capable of synthesizing mannosylerythritol lipids (MELs) (Morita et al., 2014). MELs belong to the glycolipid class of biosurfactants and are known for their outstanding interfacial and biochemical characteristics, as highlighted by Morita et al. (2015). For the Muscle model, the overall importance of features was relatively subdued, and many features contributed equally lightly in the model’s discriminatory ability. In the Stomach model, the pivotal feature was the virus *Human betaherpesvirus 7*, previously identified as an inhabitant of gastric mucosa (Gonelli et al., 2001) (Figure 4H). For the Testis model, the critical feature was the *Human endogenous retrovirus K* (HERV-K) (Figure 4I). The human genome harbors numerous copies of HERV-K, many of which retain intact open reading frames (ORFs). These ORFs are capable of being transcribed and translated, particularly during early embryonic development and in cancerous conditions (Garcia-Montojo et al., 2018). For the Bladder model, the foremost feature was the bacterium *Caldimonas thermodepolymerans*, an underexplored microorganism phylogenetically proximate to the *Comamonadaceae* group that was systematically less abundant in the samples of bladder (Figure 4J). Lastly, in the Liver model, the primary feature was *Bartonella machadoae* (Figure 4K). *Bartonella machadoea* is a bacteria belonging to *Proteobacteria* genus, and it was recently reported that the liver is inhabited mainly by proteobacteria by a gut-liver-specific axis (Broderick and Nagy, 2022).

For additional validation, the samples from the aforementioned eight tissues underwent analysis using Kaiju, a different computational method for microbial taxonomic profiling. Kaiju assigns each sequencing read to a taxon in the NCBI taxonomy by comparing it to a reference protein database. The used reference database was the microbial subset of the NCBI BLAST non-redundant protein database, encompassing bacteria, archaea, virus, fungi and microbial eukaryotes. Subsequently, GBM models for these 8 tissues were reconstructed using the taxonomic profiles generated by Kaiju. Remarkably, the performance of these models exhibited a high performance (mean AUROC ≥ 0.70 and relative mean AUPR ≥ 1.4) as depicted in Figure 4L (Supplementary File S15), indicating that regardless of the bioinformatics method analysis, these tissues present a distinctive microbial signature. The most important features of the microbial signatures found by Kaiju (Supplementary File S16) were different compared to the microbial profiles derived by AGAMEMNON. Employing different approaches for read classification, the taxonomic profiles and, respectively, the most important features of the models are differing, mainly due to the different reference databases that each tool uses for the classification of the reads and to the algorithm used in assigning the reads to each organism.

### 3.3 Heart, colon, and liver tissue preserve the tissue-specific microbial signatures in the living state

As the specimens sourced from the GTEx consortium originate from post-mortem biopsies, an investigation was conducted to examine whether the identified signatures for the eight tissues resulted from post-mortem microbial colonization or were present during the subjects’ lifetime. To address this, the eight models, comparing one tissue against the seven others, were subjected to testing using data derived from an entirely distinct project (NCBI Bioproject ID PRJEB4337). This dataset sourced from living tissues of subjects within the Swedish Biobank (Fagerberg et al., 2014). This project did not contain samples from muscle tissue and blood, so only 6 out of the 8 models (heart, colon, stomach, liver, bladder, testis models) were tested with data from living tissues. The data were processed utilizing the same pipeline as applied to GTEx data, and normalization was separately carried out on this specific distinct dataset using the CSS normalization method.

For each tissue, all 100 iterations of the 1vs7Tissues models (that were created before) were tested again using the living dataset. The obtained results were then compared with the performance of models, generated with randomly assigned tissue labels to ascertain the significant contribution of tissue origin to model performance (Supplementary File S17). Remarkably, 5 out of 6 tissues (only the model for bladder tissue did not) exhibit a statistically significant difference in AUROC and AUPR in the living dataset (*p*-value < 0.05) between the true tissue labeled model and the random tissue labeled model.

**Figure 5:**
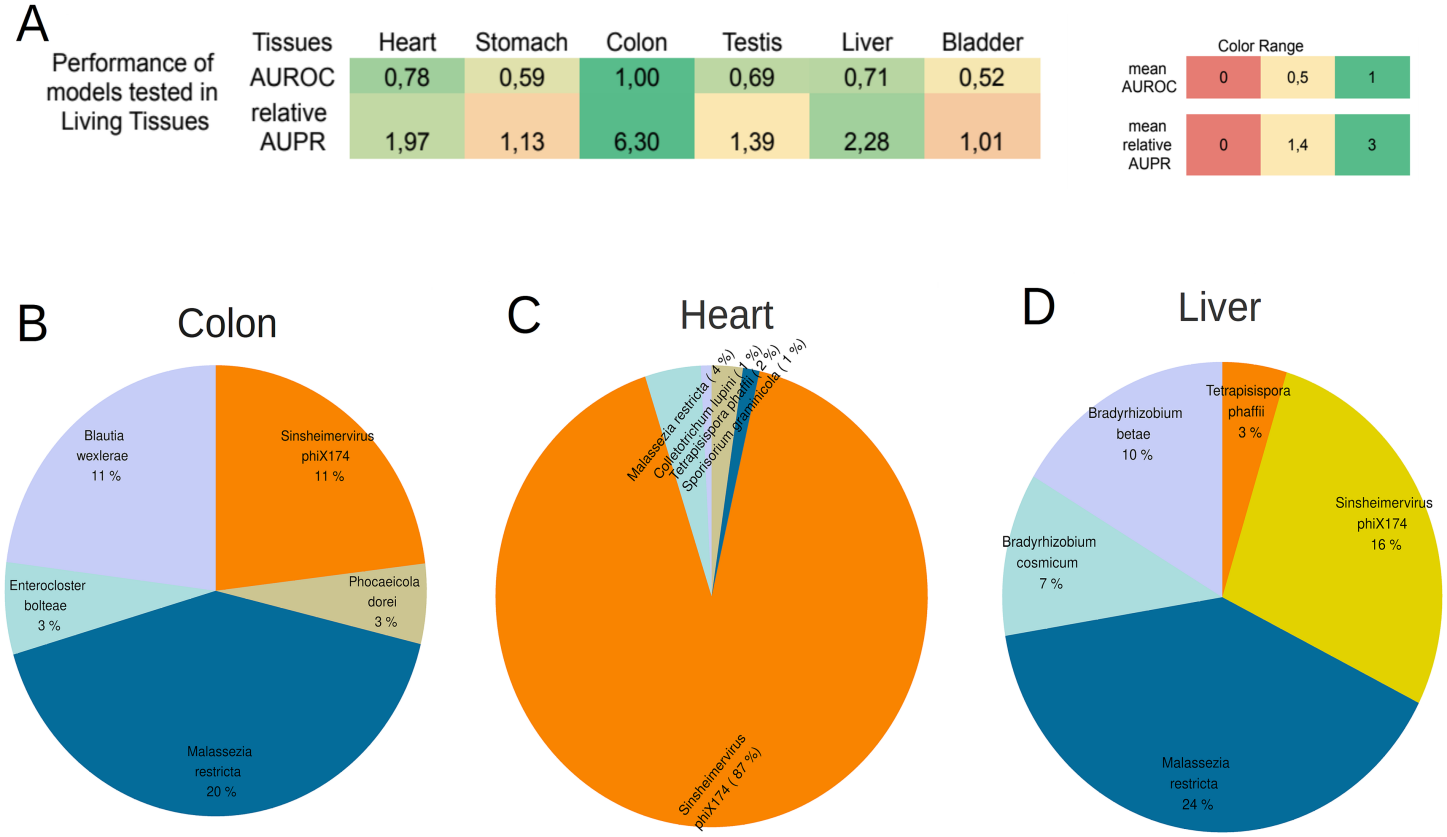
**(A)** The performance of the 8 models created from GTEx samples tested on the samples from living tissues. Colon tissue, heart and Liver presented a high AUROC and relative AUPR in the living samples, showing that the microbial signatures of these tissues are present during the lifetime of individuals too. **(B–D)** The 5 most abundant microbial species on the living samples of the colon tissue, heart, and liver. The species that are common among tissues are depicted with same color.

Among the six models, the models for Heart, Colon, and Liver tissue demonstrated robust AUROC and relative AUPR in the living dataset (AUROC ≥ 0.7 and relative AUPR ≥ 1.4) (Figure 5A; Supplementary File S18). This suggests that the microbial signatures identified in these tissues (Heart, Colon, and Liver) also exist in the healthy living state. The 5 most abundant species in the living dataset for the 3 tissues are depicted in Figures 5B–D (refer for frequencies of all microbiomes inside living tissues of the project PRJEB4337 in Supplementary File S19).

The lack of high performance for the remaining three tissues may be attributed to the likelihood that the microbial signature of these tissues undergoes significant alterations during the event of death. The high performance of the Heart, Colon, and Liver models was not observed in the models created with the taxonomic profiles derived from Kaiju (Supplementary File S20).

### 3.4 Lifestyle factors and medical history do not influence tissues’ microbiome

Additionally, the putative impact of lifestyle factors on the composition of tissue-specific microbiomes across the eight human tissues was examined. The following variables were studied: Age, BMI, Sex, Alcohol consumption, Smoking status, Ancestry. To elucidate potential associations, GBM models were constructed for each tissue to predict the specific trait. However, despite rigorous computational analyzes, the findings reveal a lack of robust predictive capability of the tissue microbiomes for these traits across all examined tissues. Detailed performance metrics for each trait within each tissue are provided in Supplementary File S21.

In addition, each tissue underwent a thorough examination to discern whether its microbial profile harbored predictive potential for various medical conditions, encompassing Alzheimer’s or Dementia, Asthma, Cerebrovascular Disease, COPD-CLRD, Depression, Diabetes Type I and II, Dialysis Treatment (Renal Failure), Rheumatoid Arthritis, Hypertension, Ischemic Heart Disease, Liver disease, Schizophrenia, Seizures, and Arthritis (as a comprehensive category inclusive of various types of arthritis). Only in the case of dialysis treatment (renal failure) could discernment be achieved between heart tissues of subjects with renal failure and heart tissues of subject without renal failure. However, for the rest of the diseases, discrimination of subjects’ diagnosis across the tissues was not feasible. Detailed performance metrics for each disease in each of the eight tissues are presented in the Supplementary File S22. Overall, the analysis of tissue microbiomes suggests a lack of inherent microbial signatures indicative of lifestyle factors or systemic disease status.

### 3.5 The utmost significance in unraveling tissue-specific microbial signatures lies within the species-level analysis

To elucidate the optimal taxonomic level for investigating microbial signatures across the eight vital tissues, we constructed GBM models utilizing microbial profiles at the genus level. AGAMEMNON supports the aggregation of microbial counts at the taxonomic level of choice. Employing microbial profiles at the genus level, we developed GBM models for the aforementioned eight tissues. GBM models based on genera exhibited slightly inferior performance compared to those derived from species-level data (refer to Supplementary File S23). Although the distinctions are subtle, the majority of the eight models demonstrated enhanced performance when analyzed at the species level; only, the bladder model exhibited superior mean AUROC and mean AUPR at the genera level.

To investigate if any other of the 20 tissues, that did not present microbial footprints at species level analysis, manifested distinct microbial signatures at the genus level, we generated GBM models utilizing taxonomic profiles at the genus level for each tissue. Only the models from the same tissues as species level models presented high performance (AUROC ≥ 0.7 and relative AUPR ≥ 1.4), most of the times slightly diminished when compared to their species-level counterparts. Only the brain tissue models did not present high performance at the genus level, but as previously shown, the species-level brain model is possibly a result of contamination (refer to Supplementary File S24).

In light of the known redundancy in the functional repertoire (i.e., genes) across diverse microorganisms, we endeavored to investigate whether tissue-specific microbial signatures predominantly arise from a tissue-specific microbial gene repertoire rather than a specific microbial taxonomy. Also, another goal was to identify tissue-specific microbial genes and pathways. To delineate the functional profile of samples, microbial reads identified by AGAMEMNON were subjected to analysis using HUMAnN 3, specifically employing translational searches against the Uniref90 database. Subsequently, GBM models for the aforementioned eight tissues were reconstructed utilizing the CSS-normalized functional profiles of the samples (refer to Supplementary File S25). Remarkably, only the Blood, Colon, and Bladder tissues exhibited high performance, with a mean AUROC ≥ 0.70 and a relative mean AUPR ≥ 1.4. In an attempt to integrate both functional and species-level information, we reconstructed GBM models using both the functional and the taxonomic profiles of the samples. However, even with this comprehensive approach, a very slight enhanced performance was attained and not in all models (see Supplementary File S26), indicating that in our scientific setup the information of functional repertoire contributes very slightly in discriminating tissues. It is crucial to note that the low performance of the functional models, could most probably be attributed to the technical characteristics of the GTEx RNA-seq data. The data, generated using a poly-A selection sequencing protocol, inherently filtered out a significant proportion of microbial RNAs.

## 4 Discussion

In each of the 28 examined tissues, a modest yet substantiated presence of transcriptionally active microbial communities was observed. Contrary to traditional notions of sterility of internal tissues, this analysis, alongside analogous investigations in other mammalian species, suggests that tissues potentially harbor a low-biomass microbiome not only in the context of disease but also in normal state. In light of recent controversies, such as the retraction of a major study on cancer tissue microbiomes (Poore et al., 2020), this analysis addresses and resolves the methodological concerns previously raised. Extending beyond pathological conditions, our analysis suggests that certain human internal tissues consistently maintain a low-biomass microbiome. This phenomenon prompts speculation that the microbiome may serve as a functional reservoir contributing to tissue well-being or act as a regulator of the immune system.

The robust discriminatory performance demonstrated by the models for eight tissues, relying solely on microbial profiles of samples, strongly suggest that the observed microbiome is not a result of random chance. Despite the impracticality of conducting *in silico* decontamination due to the uniform utilization of the same RNA quantity for each sample in the processing of GTEx samples and the absence of negative controls, a reverse analysis—implementing *in silico* contamination—illustrated the robustness of microbial signatures in eight tissues against various potential contaminations. It is imperative to clarify that the *in silico* contamination approach does not seek to substitute a laboratory analysis conducted under the most stringent sterile conditions, nor is it exhaustive in considering all conceivable contaminations. Rather, it serves as a validation step to evaluate whether contamination occurred at different stages of the analysis and to assess its potential impact on models performance. In total, eight tissues demonstrated a tissue-specific microbiome that exhibited resilience against various potential contaminations, thereby indicating the presence of a distinct microbiome in each tissue. Interestingly, this tissue-specific microbiome primarily diverges not in terms of species but predominantly in the composition of these species. The microbial signatures for heart, colon and liver tissues seem to be distinctive also in an independent dataset from healthy living tissues, supporting the presence of microbiomes inside these tissues also in a living healthy state.

In colon tissue, the most pivotal component of its microbial signature is *Flavonifractor plautii*, a prevalent bacterium in the human gastrointestinal tract recognized for its notable butyrate production (Rajilić-Stojanović and De Vos, 2014). In liver tissue, the primary microbial entity of significance is *Bartonella machadoae*. *Bartonella* species have been associated with compromised liver function (Vander Heyden et al., 2012). Lastly, the presence of the phytopathogenic fungus *Sporisorium graminicola* in cardiac tissue presents an intriguing finding. *S. graminicola* has also been isolated from human fecal samples (Natalia et al., 2023). Despite the apparent peculiarity of a phytopathogenic fungus inhabiting cardiac tissue, its consistent identification as a significant feature in both the GTEx dataset containing samples from United States and the independent test dataset from Swedish Biobank renders the possibility of mere contamination or chance occurrence highly unlikely. The ability of machine learning models to discriminate distinct microbial signatures across these three tissues in separate datasets suggests that these microorganisms are likely indigenous inhabitants of their respective tissues.

In the majority of tissues (20 out of 28 tissues), a distinct tissue-specific microbial signature was not detected. However, the absence of such a signature does not necessarily imply tissue sterility. These tissues are more likely to harbor distinct microbial signatures in species that may not have been successfully detected, possibly due to limitations imposed by the poly-A protocol or the lack of reference genomes. Indeed, the methodologies employed by GTEx protocols unequivocally revealed only a fraction of the microbial load present inside these tissues. Another plausible explanation for the absence of tissues’ microbial signatures is that the models for these tissues may not have performed optimally, as these tissues may exhibit a more versatile microbiome without a clearly defined tissue-specific signature. Lastly, for the three tissues that did not perform well in living tissues, it is conceivable that these tissues undergo a significant shift in their microbiome after death, or there may be notable differences attributable to geographical variations, as the living subjects were from a different continent than GTEx subjects.

Significantly, beyond the considerations of the poly-A sequencing protocol, metagenomics and metatranscriptomics necessitates tailored laboratory procedures for efficient DNA or RNA isolation from both gram-positive and gram-negative microorganisms. As these considerations were not initially integrated into the GTEx pipeline, it is evident that the analytical procedure failed to unveil the full spectrum of microorganisms present in the tissues. Moreover, aside from the critical role of laboratory protocols, the bioinformatics analysis protocols also play a pivotal role in discriminating and accurately describing tissue microbiomes. Two metatranscriptomics tools, Kaiju and AGAMEMNON, each employing distinct approaches for read classification, yielded different taxonomic profiles and exhibited variations in performance on the models. To advance microbiome research, it is paramount to develop specific guidelines for both laboratory and *in silico* analysis of microbiome data. This step is crucial for ensuring comprehensive and accurate insights into the diversity and composition of microbial communities within tissues.

It is crucial to consider that, in the majority of the identified microbiomes, the reads classified as belonging to these microbiomes were relatively scarce. RNA transcripts of these microbiomes were present within the tissues; however, these transcripts did not adequately cover a substantial portion of the genetic material of these microbiomes. This limitation is likely attributable to constraints imposed by the poly-A protocol. The limitations of the poly-A protocol highlight the necessity for additional investigations to validate the existence of microbiomes within tissues, rather than merely isolated microbial RNA transcripts. Another strength and limitation of the study is its focus on 28 tissues, providing substantial coverage but not fully representing the complete diversity of human internal tissues. While the exclusion of certain tissues may limit the generalizability of the findings to the entire human body, the breadth of tissues analyzed offers valuable insights into tissue-specific microbial signatures. Additionally, while the samples predominantly represent specific demographic groups, this provides a focused perspective but may limit the generalizability of the findings to more diverse populations. However, the successful application of our models to data from the Swedish Biobank, which includes individuals from a different demographic background, suggests that the findings have a degree of generalizability and broader applicability. Expanding the demographic range in future studies could further strengthen this aspect. It is pertinent to acknowledge that bioinformatic analyzes in scenarios such as the present study are subject to probabilistic constraints and, on their own, are insufficient to prove the existence of microbiomes definitively. However, they can serve as valuable tools to guide subsequent investigations, indicating tissues and organisms that warrant further scrutiny.

## 5 Conclusion

Collectively, a growing body of evidence substantiates the existence of microbial interactions with human cells in anatomical sites traditionally considered sterile. The identification of encoded signatures specific to certain tissues within normal human tissues suggests that these tissues harbor a low-biomass microbiome. This exploration signifies a paradigmatic shift, heralding an era in which we acknowledge that our bodies are not solitary entities but rather collaborative ecosystems housing diverse microbial species. We anticipate that our study will serve as a foundational resource, providing crucial guidance for future investigations and facilitating targeted laboratory validations aimed at confirming the presence of microbial communities within internal tissues.

## Data Availability

The taxonomic profiles of GTEx samples, generated using AGAMEMNON and Kaiju tools, along with their functional profiles generated via HUMAnN 3, are accessible in Zenodo (https://zenodo.org/records/12627621). Additionally, taxonomic and functional profiles for the distinctive living dataset (NCBI ID PRJEB4337) are also available on the same link, along with its metadata R object used in the analysis. Although the full metadata for GTEx samples is restricted, a subset is available at https://www.gtexportal.org/home/downloads/adult-gtex/metadata; access to the complete metadata requires approval through a dbGaP application at https://www.ncbi.nlm.nih.gov/projects/gap/cgi-bin/study.cgi?study_id=phs000424.v9.p2. All programming scripts used to download and analyze the data of the GTEx and NCBI project PRJEB4337 as well as the pipelines for normalization, in silico contamination, ML models can be found at our GitHub repository https://github.com/dianalabgr/GTEx_microbiome_analysis. These scripts are designed to directly process the summarized count data available on Zenodo. However, analysis of microbial signatures concerning phenotypic traits and medical history necessitates access to the complete metadata. For the other analytical pursuits, the open access metadata suffices; nonetheless, it is essential to accommodate variations in column names between the open access and protected access metadata files.

## Footnotes

^1^ https://gen3.theanvil.io

^2^ https://anvilproject.org/learn/reference/gtex-v8-free-egress-instructions#step-4---download-object-files

^3^ https://gtexportal.org/home/methods
