# Supplementary material for: Machine learning models reveal microbial signatures in healthy human tissues, challenging the sterility of human organs
Source: Front Microbiol. 2025 Jan 27;15:1512304. doi: 10.3389/fmicb.2024.1512304 (PMC11808598; doi:10.3389/fmicb.2024.1512304)
Supplement: Supplementary file 1 [file Data_Sheet_1.zip › Supplementary_new/Supplementary_Material_GTEx paper.docx]

***Supplementary Material***

# Supplementary Files

**Supplementary File 1.** The number of samples per tissue in GTEx dataset that were used in this analysis.

**Supplementary File 2.** The cumulative table of the reads across each step of the analysis per tissue.

**Supplementary File 3.** The species of the core microbiome (species present at least 10% of the samples of tissue) of each tissue alongside each species’ mean frequency (number of reads) for this tissue.

**Supplementary File 4.** The bacterial species of the core microbiome (species present at least 10% of the samples of tissue) of each tissue.

**Supplementary File 5.** The fungal species of the core microbiome (species present at least 10% of the samples of tissue) of each tissue.

**Supplementary File 6.** The viral species of the core microbiome (species present at least 10% of the samples of tissue) of each tissue.

**Supplementary File 7.** The archaea species of the core microbiome (species present at least 10% of the samples of tissue) of each tissue.

**Supplementary File 8.** The fungal species that are common in the core microbiome of all 28 tissues.

**Supplementary File 9.** The bacterial species that are common in the core microbiome of all 28 tissues.

**Supplementary File 10.** The viral species that are common in the core microbiome of all 28 tissues.

**Supplementary File 11.** The performance of the GBM models was evaluated using mean AUROC and mean AUPR. Each tissue's model aimed to distinguish that specific tissue from all other tissues. The mean AUROC and mean AUPR represent the average values of AUROC and AUPR across 100 iterations of the GBM model for each tissue.

**Supplementary File 12.** The comparison across the performances (mean AUROC and mean AUPR) of the GBM 1vs10-tissues models between the in silico contaminated models and the original models, using Wilcoxon’s test. For each tissue’s model, there is the mean AUROC and AUPR value for the in silico contaminated models and the original models, along with the p-value of Wilcoxon’s test for each metric.

**Supplementary File 13.** The performance of the GBM models was evaluated using mean AUROC and mean AUPR. Each tissue's model aimed to distinguish that specific tissue from the other 7 tissues. The mean AUROC and mean AUPR represent the average values of AUROC and AUPR across 100 iterations of the GBM model for each tissue.

**Supplementary File 14.** The importance score of the features for each of the 8 contamination-resilient tissues’ models. In each tab there is the feature importance score for each tissue’s model in a descending order based on their importance.

**Supplementary File 15.** The performance of the GBM models using Kaiju taxonomic profiles, was evaluated using mean AUROC and mean AUPR. Each tissue's model aimed to distinguish that specific tissue from the other 7 tissues but with the taxonomic profiles calculated using Kaiju tool. The mean AUROC and mean AUPR represent the average values of AUROC and AUPR across 100 iterations of the GBM model for each tissue.

**Supplementary File 16.** The importance score of the features for each of the 8 contamination-resilient tissues’ models using Kaiju taxonomic profiles. In each tab, there is the feature importance score for each tissue’s model in descending order based on their importance.

**Supplementary File 17.** The performance of the GBM models tested on the living dataset of PRJEB4337. Each tissue's model aimed to distinguish that specific tissue from the other 7 tissues. The mean AUROC and mean AUPR represent the average values of AUROC and AUPR across 100 iterations of the GBM model for each tissue. The random models were produced after shuffling the tissues labels from the training dataset of the GTEx data, in order to eliminate the biological meaning of the random models. A comparison of the AUROC and AUPR values for the living dataset between the random models and the original models was conducted using Wilcoxon’s test. Muscle and Blood tissues have NA values in the living dataset, as the project PRJEB4337 did not contain samples from these tissues.

**Supplementary File 18.** The performance of the GBM models created with the GTEx data, tested on the living dataset of PRJEB4337. It contains the same results as above, but without the random models. The mean AUROC and mean AUPR represent the average values of AUROC and AUPR across 100 iterations of the GBM model for each tissue. Muscle and Blood tissues have NA values in the living dataset, as the project PRJEB4337 did not contain samples from these tissues.

**Supplementary File 19.** The relative frequency of the microbial species in the living dataset for each of the 8 contamination-resilient tissues. Muscle and Blood tissues are missing, as the project PRJEB4337 did not contain samples from these tissues.

**Supplementary File 20.** The performance of the GBM models created with the GTEx data but using Kaiju taxonomic profiles, and re-evaluated using the living dataset from the project PRJEB4337. The mean AUROC and mean AUPR represent the average values of AUROC and AUPR across 100 iterations of the GBM model for each tissue. Muscle and Blood tissues have NA values in the living dataset, as the project PRJEB4337 did not contain samples from these tissues.

**Supplementary File 21.** The performance of the GBM created to distinguish each trait in each tissue of the 8 contamination-resilient tissues. In each tab, there are the results for each trait. For some tissues and some traits, there are no results as these tissues presented a small number of samples (below 20 samples) in at least one of the groups of this trait.

**Supplementary File 22.** The performance of the GBM created to distinguish each disease in each tissue of the 8 contamination-resilient tissues. In each tab, there are the results for each disease. For some tissues and some diseases, there are no results as these tissues presented a small number of samples (below 20 samples) in at least one of the groups of this disease.

**Supplementary File 23.** The performance of the GBM models created using the AGAMEMNON taxonomic profiles at genus level. Each tissue's model aimed to distinguish that specific tissue from the other 7 tissues, using the genus taxonomic profiles of samples. The mean AUROC and mean AUPR represent the average values of AUROC and AUPR across 100 iterations of the GBM model for each tissue.

**Supplementary File 24.** The performance of the GBM models created using the AGAMEMNON taxonomic profiles at the genus level. Each tissue's model aimed to distinguish that specific tissue from all the other 27 tissues, using the genus taxonomic profiles of samples. The mean AUROC and mean AUPR represent the average values of AUROC and AUPR across 100 iterations of the GBM model for each tissue.

**Supplementary File 25.** The performance of the GBM models created using the HUMAnN 3.0 functional profiles (microbial genes). Each tissue's model aimed to distinguish that specific tissue from the other 7 tissues, using the quantification of microbial genes of samples. The mean AUROC and mean AUPR represent the average values of AUROC and AUPR across 100 iterations of the GBM model for each tissue.

**Supplementary File 26.** The performance of the GBM models created using the AGAMEMNON taxonomic profiles and the HUMAnN 3.0 functional profiles (microbial genes). Each tissue's model aimed to distinguish that specific tissue from the other 7 tissues, using the species taxonomic profiles and the quantification of microbial genes of samples. The mean AUROC and mean AUPR represent the average values of AUROC and AUPR across 100 iterations of the GBM model for each tissue.

## 2 Supplementary Figures

**Supplementary Figure 1.** The UpSet plot illustrates the shared fungal species present in the core microbiome across all tissues.

**Supplementary Figure 2.** The UpSet plot illustrates the shared bacterial species present in the core microbiome across all tissues.

**Supplementary Figure 3.** The UpSet plot illustrates the shared viral species present in the core microbiome across all tissues.
