# Supplementary figures and images for: Machine learning models reveal microbial signatures in healthy human tissues, challenging the sterility of human organs
Source: Front Microbiol. 2025 Jan 27;15:1512304. doi: 10.3389/fmicb.2024.1512304 (PMC11808598; doi:10.3389/fmicb.2024.1512304)

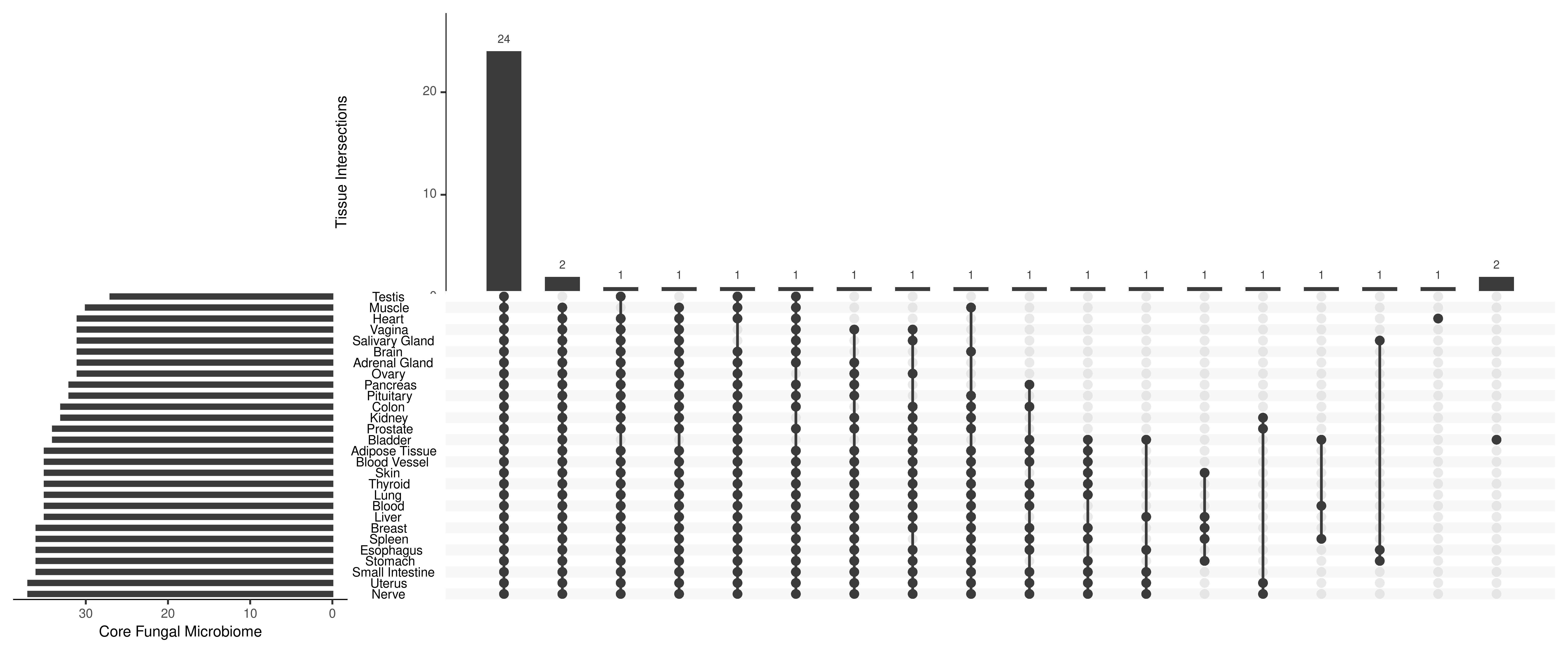

Supplement: Supplementary file 1 [file Data_Sheet_1.zip › Supplementary_new/Supplementary_Figure1.jpeg]

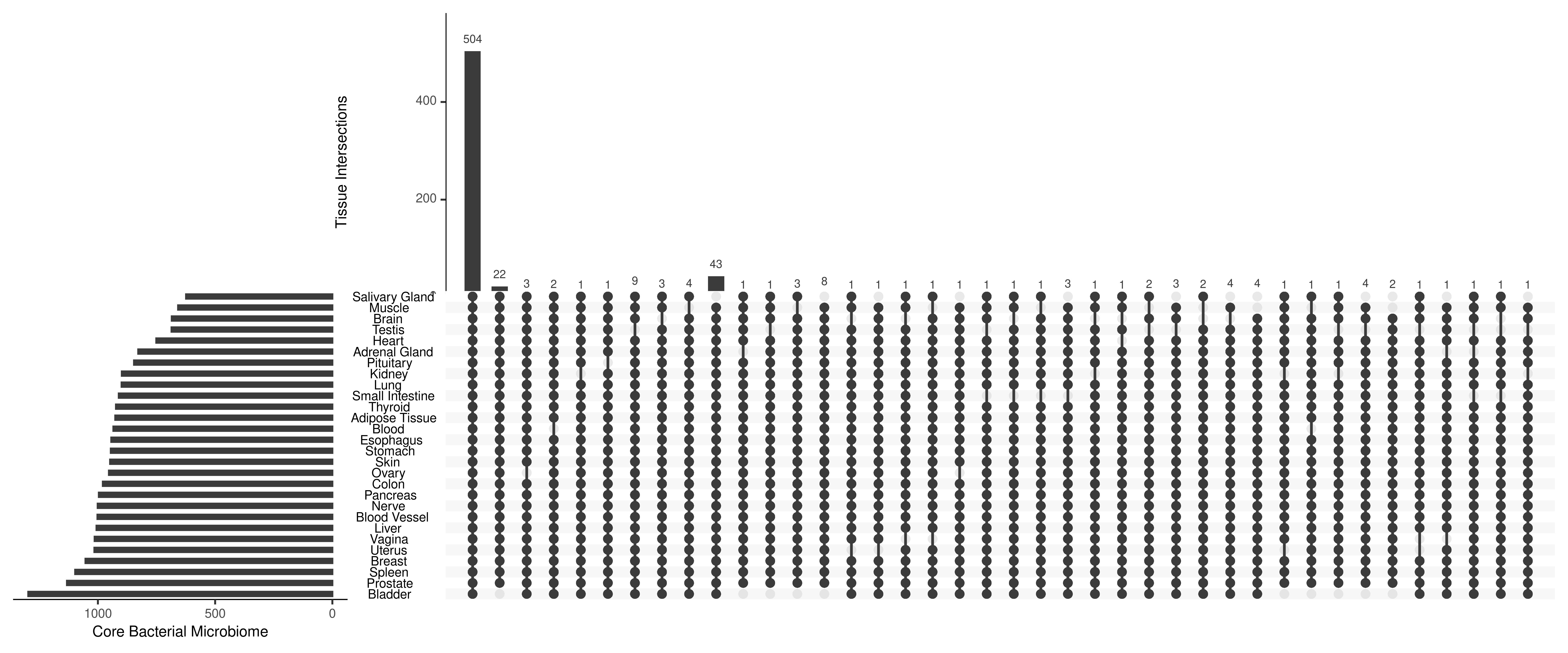

Supplement: Supplementary file 1 [file Data_Sheet_1.zip › Supplementary_new/Supplementary_Figure2.jpeg]

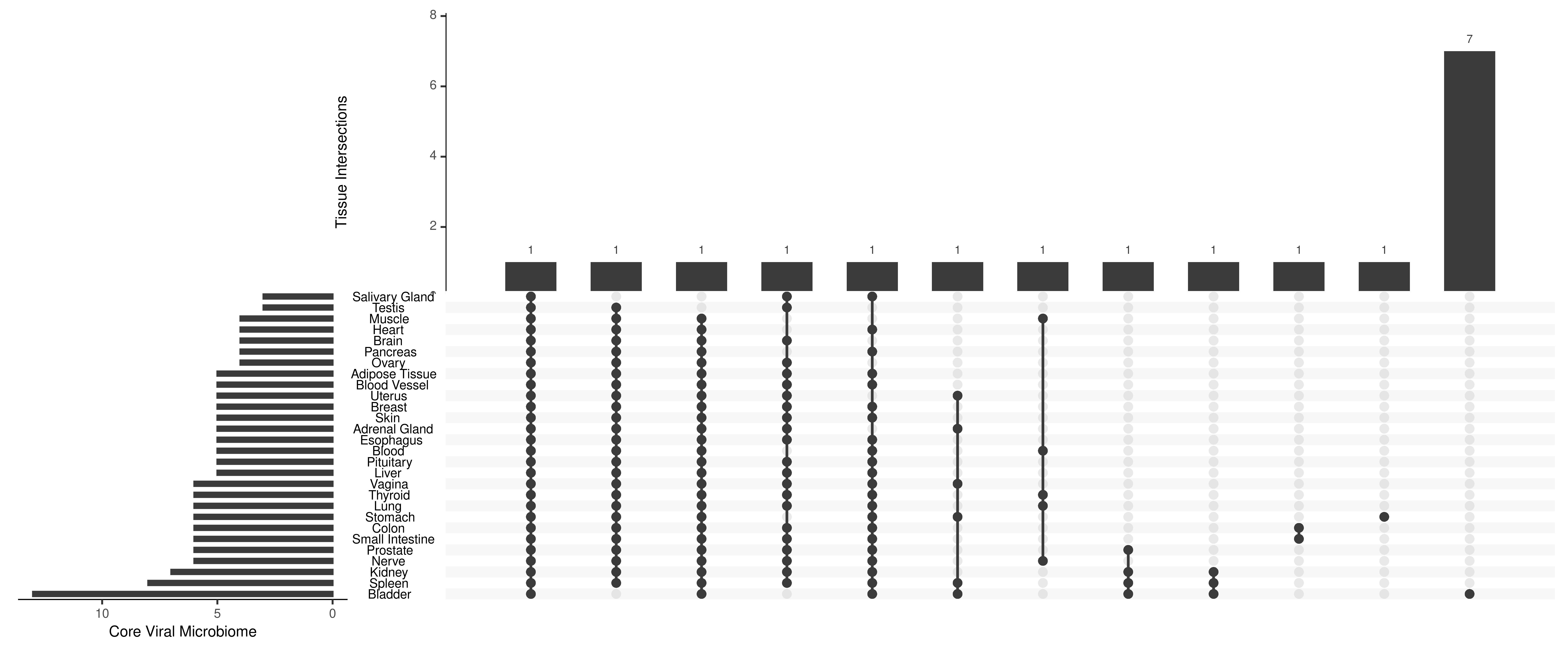

Supplement: Supplementary file 1 [file Data_Sheet_1.zip › Supplementary_new/Supplementary_Figure3.jpeg]
